# Aflatoxin Contamination of Maize, Groundnut, and Sorghum Grown in Burkina Faso, Mali, and Niger and Aflatoxin Exposure Assessment

**DOI:** 10.3390/toxins14100700

**Published:** 2022-10-12

**Authors:** Titilayo D. O. Falade, Adama Neya, Saïdou Bonkoungou, Karim Dagno, Adamou Basso, Amadou Lamine Senghor, Joseph Atehnkeng, Alejandro Ortega-Beltran, Ranajit Bandyopadhyay

**Affiliations:** 1 International Institute of Tropical Agriculture (IITA), Ibadan 200001, Nigeria; 2 Institut de l’Environnement et de Recherches Agricoles, Ouagadougou BP 8645, Burkina Faso; 3 Institut d’Économie Rurale, Bamako BP 258, Mali; 4 Institut National de la Recherche Agronomique du Niger, Niamey P.O. Box 429, Niger; 5 International Institute of Tropical Agriculture (IITA), Bukavu P.O. Box 1222, Democratic Republic of the Congo

**Keywords:** aflatoxin, susceptible crops, Sahel, exposure, cancer rick

## Abstract

Aflatoxin contamination of staple crops by *Aspergillus flavus* and closely related fungi is common across the Sahel region of Africa. Aflatoxins in maize, groundnut, and sorghum collected at harvest or from farmers’ stores within two weeks of harvest from Burkina Faso, Mali, and Niger were quantified. Thereafter, aflatoxin exposure values were assessed using per capita consumption rates of those crops. Mean aflatoxin concentrations in maize were high, 128, 517, and 659 µg/kg in Mali, Burkina Faso, and Niger, respectively. The estimated probable daily intake (PDI) of aflatoxins from maize ranged from 6 to 69, 29 to 432, and 310 to 2100 ng/kg bw/day in Mali, Burkina Faso, and Niger, respectively. Similarly, mean aflatoxin concentrations in sorghum were high, 76 and 259 µg/kg in Mali and Niger, respectively, with an estimated PDI of 2–133 and 706–2221. For groundnut, mean aflatoxin concentrations were 115, 277, and 628 µg/kg in Mali, Burkina Faso, and Niger, respectively. Aflatoxin exposure values were high with an estimated 9, 28, and 126 liver cancer cases/100,000 persons/year in Mali, Burkina Faso, and Niger, respectively. Several samples were extremely unsafe, exceeding manyfold regulatory levels of diverse countries (up to 2000 times more). Urgent attention is needed across the Sahel for integrated aflatoxin management for public health protection, food and nutrition security, and access to trade opportunities.

## 1. Introduction

Public health, food security and safety, and economic stability are negatively affected by aflatoxin contamination of crops. Aflatoxins are a group of secondary metabolites produced by several species of the ubiquitous *Aspergillus* section Flavi fungi [1]. High aflatoxin content makes foods and feeds unsafe, chronic dietary exposure to aflatoxins causes morbidity, and acute dietary exposure can result in mortality [2,3,4]. Consequently, aflatoxin levels in foods and feeds are highly regulated at very low levels, in µg/kg. There are four major aflatoxins: B1, B2, G1, and G2. Aflatoxin B1 (AFB1) is the most toxic of the four. Naturally occurring mixes of aflatoxins are categorized as a Group 1 carcinogen, the highest category given by the International Association for Research on Cancer [5]. Since AFB1 is a genotoxic carcinogen, there is no tolerable daily intake for aflatoxin. However, exposure to 1–2 ng/kg bw/day is estimated to be of little to no risk for populations in regions where the hepatitis B virus (HBV) is not endemic [6,7], but significant threats exist in regions where daily exposure is acute (e.g., 20–120 µg/kg bw/day) and where HBV is endemic [8]. Aflatoxin exposure, including sub-acute levels, are directly linked to hepatocellular carcinoma (HCC) and are associated with other cancers, immunosuppression, and stunting in children [9,10]. In animals exposed to aflatoxin contaminated feeds, intestinal, kidney, and/or liver disorders, reduced productivity, and mortality can occur depending on aflatoxin concentration and age of the specimen, among other factors [11,12,13]. The toxins commonly accumulate in susceptible crops and unfortunately many people, particularly in developing countries, are exposed to the toxins beginning in utero and then throughout their life [10].

For most foods, regulatory limits for total aflatoxins are set at below 4 µg/kg and 20 µg/kg in the European Union and the United States, respectively. Several African countries have also adopted regulatory limits between 4 and 20 µg/kg (www.aflatoxinpartnership.org (accessed on 20 April 2022)), although this is enforced more for crops to be exported than for locally consumed crops, including home-grown crops. Crops exceeding regulatory limits have reduced access to domestic and international premium markets, and this contributes to decreased economic empowerment [14]. Crop products not meeting standards typically enter local markets where regulations are difficult to be enforced [15]. Consequently, there is increased risk of higher aflatoxin dietary exposure in local food systems. Unfortunately, this tends to be the norm in several low- and medium-income countries (LMICs), including within sub-Saharan Africa (SSA).

Aflatoxins contaminate a wide range of foods, including staples of many LMICs, such as maize, groundnut, sorghum, and traditional foods [16,17,18,19]. Sorghum, a drought-tolerant crop, previously reported to be less susceptible than other cereals [20], has been reported in recent years to contain high aflatoxin levels [18]. Maize and groundnut are continuously reported to have high aflatoxin levels, including in the Sahel, a semi-arid region immediately south of the Sahara that cuts across Sudan to Senegal along the East-West axis [16]. Although introduced from the Americas, maize and groundnut have become dietary staples in the Sahel and in SSA in general. Consequently, as aflatoxin-susceptible staples, a significant proportion of populations in the Sahel are continuously exposed to aflatoxins. Furthermore, climatic shifts are increasing crop stress in SSA [21], which is already within the zone of high risk of perennial exposure to mycotoxins, thus, further increasing aflatoxin biosynthesis by the causative fungi [22].

There are several *Aspergillus* spp. capable of producing aflatoxins, but *A. flavus* and *A. parasiticus* are the most common causal agents of contamination. The former produces only B aflatoxins at variable levels, while the latter produces consistently high levels of both B and G aflatoxins^1^. *A. flavus* is composed of the L and S morphotypes. These morphotypes differ in several characters, but the most obvious is that the L produces few large (>400 µm) sclerotia, while the S produces numerous small (<400 µm) sclerotia [23]. The S morphotype consistently produces high B aflatoxin concentrations, while members of the L morphotype produce variable B aflatoxin levels with some completely lacking abilities to produce aflatoxins due to defects in genes responsible for aflatoxin biosynthesis [24]. Non-aflatoxin-producing members of the L morphotype are being used in aflatoxin biocontrol programs in several countries, including in SSA [25]. Across the globe, fungi resembling the S morphotype of *A. flavus* have been recovered from a variety of substrates. Using phylogenetic analyses, those fungi have been assigned to diverse species, including *A. aflatoxiformans*, *A. austwickii*, *A. cerealis*, *A. minisclerotigenes*, *A. pipericola*, and *A. mottae* [1]. Many of these spp. occur in SSA, and some of them produce both B and G aflatoxins. Their correct assignment to the species level is still expensive (particularly if thousands of isolates are examined) and therefore, are sometimes referred to as S morphotypes, S strains, or S_BG_ strains if producing both B and G aflatoxins.

In the current study, aflatoxin contamination in the staple crops sorghum, groundnut, and maize was investigated in major production zones of Burkina Faso, Mali, and Niger. The objectives of this work were to identify aflatoxin hotspot areas and the risk that aflatoxin contaminated crops may pose to populations in the three Sahelian countries. We found elevated aflatoxin levels in some samples which were mostly collected within 1–2 weeks of harvesting. This is indicative of pre-harvest aflatoxin contamination, which can worsen under sub-optimal storage conditions. The results indicate that populations in those countries are at high risk of aflatoxin-associated diseases. Prompt, effective technical, institutional, and policy actions are needed to reduce threats that aflatoxins pose to food security and safety, public health, and trade in the Sahel.

## 2. Materials and Methods

### 2.1. Sample Collection

Maize, groundnut, and sorghum samples were collected from farmers’ fields or stores (within 1–2 weeks of harvesting) in Burkina Faso, Mali, and Niger. All samples were collected in the dry season and transported in paper bags. Sorghum samples were collected in Mali and Niger only. Sample collection and processing is described for each of the countries. Samples (3 to 5 kg) were collected in major crop production areas, labeled, and transported to the respective plant pathology laboratories of the national agricultural research institutes. Maize and groundnut samples were threshed with a thresher, and sorghum was threshed manually with a stick at the farmers’ locations.

**Figure 1:**
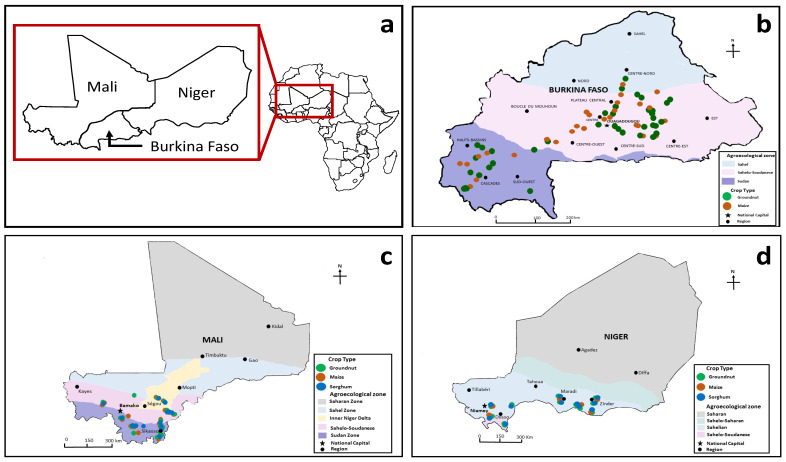
Map indicating the locations within Africa of the three countries (**a**) and locations of fields cropped to maize, groundnut, and sorghum that were sampled in Burkina Faso in 2010 (**b**), Mali in 2017 and 2018 (**c**), and Niger in 2019 (**d**). We used the leaflet R-package (https://leafletjs.com/reference-1.7.1.html (accessed on 1 April 2022)) to create the maps in (**a**–**d**).

*Burkina Faso.* In 2010, 62 maize and 53 groundnut samples were collected across Burkina Faso in various provinces of three agroecological zones (AEZs) (Figure 1). After collection, samples were air dried in the shade for about 10 days before they were transported to the Institut de l’Environnement et de Recherches Agricoles (INERA) Plant Pathology Laboratory at Farako-Bâ, Bobo Dioulasso, Burkina Faso.

*Mali.* There were 112 maize, 91 groundnut, and 85 sorghum samples collected during the sampling period (December 2017–January 2018) from the regions of Kayes, Koulikoro, Ségou, and Sikasso (Figure 1). Grain samples were air dried in the shade for about 10 days before they were transported to the Institut d’Economie Rurale (IER) Plant Pathology Laboratory at Sotuba, Bamako, Mali.

*Niger.* In 2019, 123 maize, 149 groundnut, and 145 sorghum samples were collected from the regions of Dosso, Maradi, Niamey, Tillabéri, and Zinder (Figure 1). Grain samples were air dried in the shade for about 10 days before they were transported to the Institut National de la Recherche Agronomique du Niger (INRAN) Plant Pathology Laboratory at Niamey, Niger.

### 2.2. Sample Processing

At the respective national laboratories, each sample was individually homogenized (by mixing grains in individual sample bags by hand) and divided into two equal portions. One half of the samples were sent to IITA Pathology and Mycotoxin Laboratory in Ibadan, Nigeria by airfreight, and the other was kept as a backup. Sample dispatch was done after required permits were obtained. Nigeria Agricultural Quarantine Service (NAQS) provided import permits. Thereafter, phytosanitary certificates from the three countries were obtained. When samples arrived at Ibadan, they were cleared at NAQS and analyzed. The period from sample collection to analysis was within two weeks in each of the countries. Sampling was done in the dry season. After arrival at IITA, maize and sorghum samples were ground using a coffee mill grinder (Bunn-o-Matic Corporation, Springfield, Oregon, IL, USA), while groundnut samples were ground using a high-speed laboratory blender (Waring Commercial, Springfield, MO, USA). This achieved a particle size of <1 mm. Ground samples were thoroughly mixed and placed in sealed labeled plastic bags prior to cold storage at 4 °C until further analysis. The grinder and blender were thoroughly washed between samples with 80% ethanol to prevent cross-contamination.

### 2.3. Aflatoxin Quantification

For groundnut, 20 g of the milled sample were combined with 100 mL 80% methanol [26], while for maize and sorghum 20 g of the milled samples were combined with 100 mL 70% methanol [27]. The mixtures of each crop sample were shaken on a Roto-Shake Genie (Scientific Industries, Bohemia, NY, USA) for 30 min at 400 rpm and then filtered through Whatman No. 1 filter paper (Whatman Intl. Ltd., Maidstone, England). Then, aflatoxins were extracted, developed on thin layer chromatography plates alongside aflatoxin standards using diethyl ether:methanol:water (96:3:1) mixture in a development chamber and quantified with a scanning densitometer coupled with winCATS software, as described previously [28]. Total aflatoxins (TAF) were calculated by adding aflatoxins B1, B2, G1, and G2.

### 2.4. Data Analysis

TAF values were log-transformed [y = log_10_ (1 + TAF in µg/kg)] to normalize variances. In each country, aflatoxin contents in individual crops were examined across regions, and aflatoxin contents among crops were contrasted within regions. Data were subjected to analysis of variance, and means were separated using Student–Newman–Keuls test (α = 0.05). For Burkina Faso, SGS (Southern Guinea Savannah) AEZ was not included in the analysis because of the low number of samples. Similarly, for Niger, sorghum samples from the Niamey region were not included. All statistical analyses were conducted using SAS software v9.1 (SAS Institute, Cary, NC, USA).

### 2.5. Assessment of Exposure

To assess risks posed by dietary exposure to aflatoxins to Nigerien, Malian, and Burkinabé populations, the probable daily intake (PDI), margin of exposure (MOE), and HCC risk rates were estimated using the formulas below for the regions where samples were collected for maize and sorghum separately, as in other studies [29,30,31,32].

PDI (ng/kg bw/day) = (*μ* × C)/bw

where *μ* = mean AFB1; C = daily per capita consumption of grain in country; bw = average body weight of an adult in kg.

MOE = BMDL/PDI

where BMDL = benchmark dose lower limit and was set at 170 ng/kg bw/day [33].

AP (average potency) = (0.3 × proportion of HBsAg-positive prevalence rate) + (0.01 × proportion of HBsAg-negative prevalence)

where HBsAg = positive hepatitis B surface antigen.

HCC risks = PDI × AP

where PDI and AP are as previously calculated.

The daily per capita consumption (DPPC) of sorghum and maize was obtained from published reports [33,34,35,36]. A DPCC of sorghum of 309 g, 548 g, and 548 g for Mali, Niger, and Burkina Faso, respectively, was used. The DPCC used for maize was 118 g, 216 g, and 468 g for Mali, Niger, and Burkina Faso, respectively. The DPCC of groundnut was not available, and HCC risk for groundnut consumption was not estimated. The average bw used for all three countries was 56 kg from an estimated average for both males and females previously reported for Mali [37]. The prevalence of positive HBsAg used to calculate the AP was 20% in Mali, 16% in Niger [38], and 11% in Burkina Faso [39]. AFB1 values were used to calculate exposure of Malian and Nigerien populations. However, 50% of TAF was used as the estimate for AFB1 values for Burkinabé samples which were then applied to risk assessment calculations [40]. A minimum exposure level of 0.017 ng/kg bw/day was used based on European Food Safety Authority’s advice that exposure above this is considered a public health concern [41].

## 3. Results

Aflatoxin concentrations varied among crops and within locations in the three Sahelian countries. Generally, the average aflatoxin levels were higher in Niger, followed by Mali and Burkina Faso. Maize and groundnut generally had higher aflatoxin levels compared to sorghum in Niger and Mali. More detailed results are reported per country below.

## 4. Burkina Faso

*Aflatoxin analyses.* TAF in maize and groundnut varied among the examined AEZs. When individually comparing maize and groundnut between AEZs, there were no significant (*p* > 0.05) differences in aflatoxin content (Table 1). When comparing both crops in individual AEZs, maize had higher (*p* < 0.05) aflatoxin content than groundnut in both cases (Table 1). For maize, aflatoxin content reached 517 µg/kg in a sample from Komandjari, while 926 µg/kg were detected in a groundnut sample from Kourwéogo.

*Assessment of exposure.* Aflatoxin exposure was high. The PDI of aflatoxins from the consumption of maize ranged from 29 ng/kg bw/day in Comoé to 672 ng/kg bw/day in Komandjari (Table 2). The MOE ranged from 0.3 to 2.9. HCC risks ranged from 1.2 cases/100,000 persons/year (CPY) to 28.6 CPY (Table 2).

## 5. Mali

**Figure 2:**
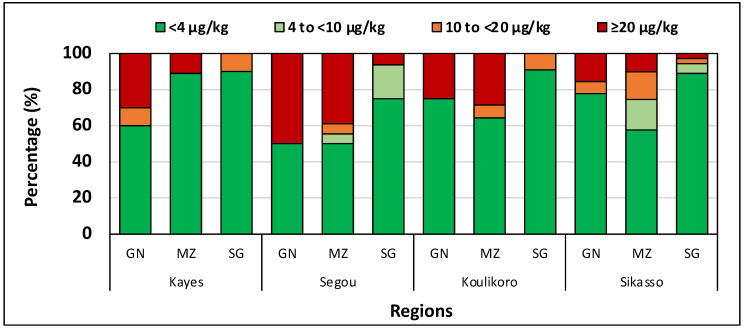
Percentage of samples containing different aflatoxin concentration (µg/kg) categories in groundnut, maize, and sorghum sampled in Kayes, Koulikoro, Ségou, and Sikasso, Mali. GN: groundnut; MZ: maize; SG: sorghum.

*Aflatoxin analysis.* Aflatoxin concentrations varied among the crops and regions (Table 3). However, there were no significant differences within a crop across regions. Sorghum in all regions had lower (*p* < 0.05) aflatoxin content than maize and groundnut. Total aflatoxin concentrations reached 1848 µg/kg in a maize sample from Ségou, 1245 µg/kg in a groundnut sample from Ségou, and 35 µg/kg in a sorghum sample from Sikasso. Up to 50% of the samples in each region, particularly groundnut, contained aflatoxin levels exceeding the regulatory limit of 20 µg/kg (Figure 1). Across all regions, most sorghum had relatively low aflatoxin levels, and 75% to 90% did not exceed the stringent EU regulatory levels of 4 µg/kg (Figure 2). Variances between individual samples were lowest in sorghum compared to maize and groundnut (Table 3).

*Assessment of exposure.* Aflatoxin exposure differed among regions (Table 4). The PDI of aflatoxins was higher through consumption of maize than of sorghum in all regions except Ségou where sorghum had a higher PDI than maize (133 vs. 58 ng/kg bw/day). The PDI from maize was highest in Kayes (69 ng/kg bw/day) and least in Sikasso (6 ng/kg bw/day). The least PDI due to sorghum consumption was in Sikasso and Kayes at 2 ng/kg bw/day. HCC risks due to consumption of maize ranged from 0.4 CPY (Sikasso) to 4.7 CPY (Kayes) and from 0.2 CPY (Kayes, Koulikoro, Sikasso) to 9.1 CPY (Ségou) due to consumption of sorghum.

## 6. Niger

**Figure 3:**
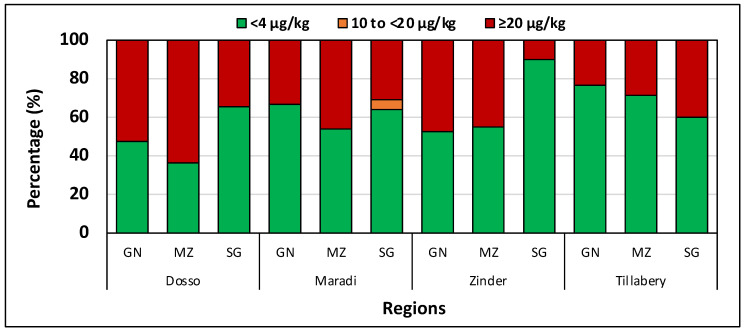
Percentage of samples containing different aflatoxin concentration (µg/kg) categories in groundnut, maize, and sorghum sampled in Dosso, Maradi, Zinder, and Tillabéri, Niger. GN: groundnut; MZ: maize; SG: sorghum.

*Aflatoxin analysis.* Aflatoxins were detected in all crops in all regions within Niger (Table 5). There were detectable aflatoxins in 41% of the samples. All contaminated samples had over 10 µg/kg TAF, and 39% had more than 20 µg/kg TAF. Aflatoxin concentrations in sorghum, maize, and groundnut, reached 1988 µg/kg, 5886 µg/kg, and 8593 µg/kg, respectively. Generally, aflatoxin concentrations in sorghum were lower than concentrations in maize and groundnut (Table 5). Mean aflatoxin levels were significantly (*p* < 0.05) higher in Dosso for maize (659 µg/kg) compared to other regions. For groundnut, aflatoxin content was statistically similar in all regions except Tillabéri where levels were significantly (*p* < 0.05) lower. Nevertheless, the average aflatoxin content in Tillabéri was still high (90 µg/kg; Table 5). The aflatoxin content in sorghum was lowest (*p* < 0.05) in Maradi. Aflatoxin concentrations in sorghum were lower (*p* < 0.05) than those in maize and groundnut in Zinder, Maradi, and Tillabéri (Figure 3). Maize and groundnut from Tillabéri contained safer aflatoxin levels than the same crops in other regions (Figure 3).

*Assessment of exposure.* The dietary exposure to aflatoxins was very high in Niger, ranging from a PDI of 310 ng/kg bw/day in Maradi to 2100 ng/kg bw/day in Dosso (Table 6) from maize consumption. Consequently, low MOE were recorded, ranging from 0.1 to 0.5. HCC risks from maize consumption were high ranging from 17.7 to 119.7 CPY. The PDI from sorghum consumption was also high and ranged from 253 ng/kg bw/day in Maradi to 2221 ng/kg bw/day in Niamey. Consequently, the MOE was also very low (range = 0.1 to 0.7), and HCC risks due to sorghum consumption ranged from 14.4 CPY to 126.6 CPY across regions (Table 6).

## 7. Discussion

The current study evaluated aflatoxin concentrations in sorghum, groundnut, and maize grown in Burkina Faso, Mali, and Niger; the samples were collected in different years. The results discussed reflect the prevalence of aflatoxins in the different years collected during the dry season. Aflatoxin contamination would vary across years, seasons, and with variations in environmental and management conditions. Concentrations within and among crops and countries varied (Table 1, Table 3 and Table 5). Over 44% of sorghum, groundnut, and maize samples were contaminated with aflatoxins, and 30.6% of those contained levels above 20 µg/kg, the regulatory limits in the U.S. (Table 2, Table 4 and Table 6). There were some cases in which extremely high aflatoxin levels were recorded and that put the population at high exposure, particularly in Niger. Sorghum is generally regarded to be less susceptible to aflatoxin contamination compared to other crops [20]. Due to its tolerance to drought, it is also an important crop for food security. However, results from the current study, although revealing that it was the less susceptible to contamination compared to maize and groundnut, indicate that it requires integrated strategies to manage aflatoxins.

The high levels of aflatoxins in many of the examined samples continue to demonstrate that farmers in the three Sahelian countries need aflatoxin management interventions at both the pre- and post-harvest stages (Table 1, Table 3 and Table 5). Aflatoxin contamination occurs when toxigenic members of *Aspergillus* section *Flavi* infect crops, and the right conditions for contamination occur. Aflatoxin-producing fungi reach crops at the pre-harvest stage from propagules that are present in organic material on the fields as debris or other crop materials. During storage, high levels of aflatoxins occur when conducive conditions of temperature, humidity, and sub-optimal storage converge [42]. Moreover, populations in these countries get most of their dietary needs (over 60%) from low diverse diets that include mostly cereals, roots, and tubers [43,44], and many of those staples are prone to aflatoxin contamination. This suggests that there is a high exposure to aflatoxins, as demonstrated in the current study. Other studies have reported high prevalence of aflatoxin contamination and/or exposure in Burkina Faso, Mali, and Niger. In Burkina Faso, it has been reported that up to 50% of maize samples were contaminated with aflatoxins [19], up to 135 µg/kg of aflatoxin were found in infant formula made from locally sourced grains, and up to 258 µg/kg in maize and rice [45]. Milk in Burkina Faso, on the other hand, appears not to be an important source of exposure to aflatoxin (aflatoxin M1; found in milk produced by livestock that ingested aflatoxin contaminated feeds) based on preliminary data [46], and this can be related to cattle being mostly grass-fed with little supplementation with cereal brans and crop residues [47]. In a study in Mali, aflatoxins were prevalent in 100% of the samples collected during the rainy season [48]. Other studies reported high contamination of grain samples at harvest from Mali (about 60%) with levels exceeding 4 µg/kg (the EU regulatory limit) that increased during storage [16]. In Niger, maize production is not sufficient, and therefore, maize has to be imported from neighboring West African countries (e.g., Benin, Burkina Faso, and Nigeria); a recent study reported that some maize offered in Nigerien markets contain high aflatoxin levels, and this was associated with poor post-harvest management, including high insect infestation [49]. Also in Niger, local production of groundnut has been reported to be affected by pre-harvest aflatoxin contamination attributed to stress conditions and agronomic practices [50].

Safety of staples must be improved in the three Sahelian countries. In addition, improvement of the economies to enable citizens to have sufficient economic power to diversify their diets is needed. Managing aflatoxins can help to address both needs. Food safety is improved if crop quality is protected, including successfully reducing aflatoxin contamination. Also, with improved food safety, household income may improve as health burdens caused by aflatoxin exposure DALYs are reduced [51]. Furthermore, capacity to engage in international trade is enhanced and income is improved when crops meet regulatory requirements of importing countries [14,52]. Of course, access to premium markets to producers of aflatoxin-safe crops is critical for this to be realized.

Aflatoxin levels in some samples were very high across regions in all the crops in the three countries (Table 1, Table 3 and Table 5). There was a high proportion of samples exceeding tolerance thresholds (Figure 1 and Figure 2). The samples were collected immediately after harvest or within 1–2 weeks of harvesting, which suggests that aflatoxins accumulated at the pre-harvest stage. Several samples contained aflatoxin levels extremely unsafe for human and animal consumption. In countries where food and feed grade systems exist and are operational, breeding and finishing cattle can be fed with maize and groundnut containing less than 100 µg/kg and 300 µg/kg, respectively [53]. Several samples in the current study greatly exceeded those levels. In the EU, aflatoxins are regulated at less than 4 µg/kg, but levels in some crops averaged hundreds of times more than that level. There were some samples with well over 900 µg/kg aflatoxin in the three countries (Table 1, Table 3 and Table 5) and up to 8500 µg/kg aflatoxin in Niger. Either highly toxigenic fungi contaminated those crops at alarming levels in the field or the short storage period (1–2 weeks) and most likely in sub-optimal conditions was sufficient to allow toxigenic fungi to produce such dangerous concentrations. Although these grains are seldom consumed raw and would undergo processing, these levels of exposure pose a risk. Processes, such as boiling and roasting, would mildly reduce aflatoxin levels as the toxins are heat stable.

Aflatoxins do not have a tolerable limit due to their genotoxic properties, and no consensus has been reached on a tolerable daily intake. In EU countries, aflatoxin levels are required to be as low as reasonably achievable [33]. In many African countries there are regulatory limits set but hardly enforced for domestic markets. In the current study, the detected high aflatoxin levels in staple crops are a serious public health concern since these crops constitute a major source of energy. Regressive child development has been associated with high dietary exposure to aflatoxins in weaning foods, breastmilk, and pre-birth through transplacental exposure [54,55,56,57,58,59]. Dietary exposure to aflatoxins has also been associated with disorders in spermatogenesis [60]. There is an established causal relationship between chronic dietary exposure to aflatoxins and HCC, particularly in regions where the levels of exposure to HBV is high, resulting in 30 times higher HCC risk [61,62]. The high exposure to unsafe aflatoxin levels (Table 2, Table 4 and Table 6) requires urgent attention by all relevant stakeholders. Up to 95% of the contaminated samples contained AFB1 proportions that were above 50% of the total aflatoxins. This is a typical pattern for samples contaminated by *A. flavus* and poses a high risk of HCC, especially as AFB1 is the most carcinogenic of the four major aflatoxins [5].

In all three countries, the aflatoxin exposure threshold (0.017 ng/kg/day) was surpassed more than 14 times (Table 2, Table 4 and Table 6). Among all types of cancer, HCC is the fourth most common in SSA, with aflatoxins contributing to 10% of these cancers [63]. This estimate is possibly conservative as HBV is considered to contribute to 70% of HCC and may not have been combined with aflatoxin exposure but considered independently. It is imperative that aflatoxin exposure is considered as a high priority for intervention in SSA countries for public health, food, nutrition, and income security in the sub-region. There are several technologies and practices available for aflatoxin management in these crops [64,65,66]. Effective technical, institutional, and policy options need to be converged to reduce the incidence of aflatoxins in these countries to protect populations and enhance international trade. Grain samples in this study were collected in three different years—2010, 2017, and 2019. Whereas there would be variations in the Sahelian environmental conditions across these years in these countries, the data collected also presents a persistent aflatoxin contamination problem in these crops regardless of the sampling year. Although not reported in the current study, the samples were also used to characterize the aflatoxin-producing fungal communities associated with these crops. There were a large number of atoxigenic isolates of *A. flavus* identified, and these could be used as biocontrol agents to limit aflatoxin contamination (unpublished). Some atoxigenic isolates from Burkina Faso have been characterized, and the type of lesions in the aflatoxin biosynthesis gene cluster causing loss of aflatoxin-production ability have been described [67]. Currently, atoxigenic isolates of *A. flavus* used as active ingredients of aflatoxin biocontrol products in SSA successfully reduce aflatoxin contamination when used at the pre-harvest stage [25,68]. Such strategy used at scale could help reduce aflatoxin levels and exposure in Burkina Faso, Mali, and Niger and would contribute to economic growth through trade in domestic and international aflatoxin-conscious markets. The results presented in this study reflect the prevalence of aflatoxins in raw samples collected at those locations and times. There is often variability in aflatoxin contamination across seasons and locations. Regular up-to-date monitoring is important for current risk assessments and for guidance to policy makers towards the institution of risk management systems pre-harvest and post-harvest.

## 8. Conclusions

Aflatoxin management of groundnut, maize, and sorghum in Burkina Faso, Mali, and Niger is critically needed to attain food security and food safety. For the first time, we report aflatoxin exposure assessments in these Sahelian countries. The risk of exposure to aflatoxins through dietary consumption was high in all three countries with the highest risk in Niger, followed by Mali, and Burkina Faso. Aflatoxin-contaminated staples are consumed more frequently than other crops, and this increases the risk of exposure. The higher levels of aflatoxins in maize and the higher dietary intake of the crop in the countries constitute a higher population risk to HCC because of the synergistic interaction of aflatoxins and HBV. It is important to note that the risk is higher for individuals who already have an underlying illness further reducing their immunity. The problem becomes worse in food insecure situations where people suffer malnutrition and/or have reduced access to high-quality foods. It is therefore critical that urgent efforts are put in place to manage the crises caused by aflatoxins in these countries. Aflatoxin contamination must be addressed from the pre-harvest stage through post-harvest practices up to processing and consumption. Doing so would contribute to better food security, food safety, reduced public health problems, and economic security.

## Figures and Tables

**Table 1:** Aflatoxin concentration (µg/kg) in maize and groundnut samples collected in 16 provinces in three AEZs of Burkina Faso in 2010. AEZ: agroecological zone; NGS: Northern Guinea savannah; SS: Sahel savannah; SGS: Southern Guinea savannah. ND: not detected. Same uppercase letters indicate means that are statistically similar in the columns (i.e., between AEZ), while same lowercase letters indicate means that are statistically similar in a row (i.e., between crops in the same AEZ). Means of log aflatoxin concentrations of NGS and SS were separated using Student–Newman–Keuls test (α = 0.05); SGS was not included in the statistical analysis because of the low number of samples.

	Total Aflatoxin (µg/kg)											
	Maize						Groundnut					
AEZ	n	Min	Max	Mean	Median	Variance	n	Min	Max	Mean	Median	Variance
NGS	21	3	140	20.1 Aa	11.8	836	15	ND	53	11.2 Ab	7.1	231
SS	34	2	517	54.3 Aa	17.9	12,930	33	ND	926	47.7 Ab	8.8	27,239
SGS	7	ND	12	7.7	8.2	15	5	ND	22	13.7	13.9	73

**Table 2:** Risk assessment of aflatoxin dietary exposure from maize sampled from different AEZs in Burkina Faso. AEZ: agroecological zone; NGS: Northern Guinea savannah; SS: Sahel savannah. PDI: probable daily intake. MOE: margin of exposure. HCC rates: hepatocellular carcinoma rates (cancer/year/100,000 persons).

AEZ	Provinces	PDI (ng/kg bw/day)	MOE	HCC Rates
NGS	Balé	76	2.2	3.2
	Boulgou	44	3.9	1.9
	Houet	143	1.2	6.1
	Kénédougou	79	2.2	3.4
	Kouritenga	58	2.9	2.5
SGS	Léraba	34	5.0	1.5
	Comoé	29	5.9	1.2
SS	Bazéga	56	3.1	2.4
	Boulkiemdé	84	2.0	3.6
	Gnagna	255	0.7	10.8
	Gourma	66	2.6	2.8
	Kadiogo	102	1.7	4.3
	Komandjari	672	0.3	28.6
	Kourwéogo	156	1.1	6.6
	Oubritenga	432	0.4	18.3
	Sanmatenga	59	2.9	2.5

**Table 3:** Mean aflatoxin concentrations (µg/kg) in maize, groundnut, and sorghum sampled from farmers’ fields across different regions in Mali. Same uppercase letters indicate means that are statistically similar in a column (i.e., across regions); same lowercase letters indicate means that are statistically similar in a row (i.e., across crops). Means of log aflatoxin concentrations were separated using Student–Newman–Keuls test (α = 0.05). ND: not detected.

Region	Total Aflatoxin (µg/kg)																	
	Maize						Groundnut						Sorghum					
	n	Min	Max	Mean	Median	Variance	n	Min	Max	Mean	Median	Variance	n	Min	Max	Mean	Median	Variance
Kayes	9	ND	1076	119.5 Aa	ND	128,586	10	ND	939	115.4 Aa	ND	86,552	10	ND	11	1.1 Ba	ND	12.8
Koulikoro	14	ND	159	27.7 Aa	ND	3190	12	ND	210	33.8 Aa	ND	4530	11	ND	16	1.4 Ba	ND	23.0
Ségou	18	ND	1849	156.3 Aa	3.1	189,468	14	ND	1245	124.4 Aa	27.3	106,065	16	ND	27	2.9 Ba	ND	49.0
Sikasso	59	ND	188	12.6 Aa	ND	1147	44	ND	1235	58.6 Aa	ND	47,177	36	ND	35	1.5 Ba	ND	36.0

**Table 4:** Risk assessment of aflatoxin exposure from maize and sorghum crops samples from different regions in Mali. PDI: probable daily intake. MOE: margin of exposure. HCC rates: hepatocellular carcinoma rates (cancer/year/100,000 persons).

	Maize			Sorghum		
Region	PDI (ng/kg bw/day)	MOE	HCC Rates	PDI (ng/kg bw/day)	MOE	HCC Rates
Kayes	69	2.5	4.7	2	74.6	0.2
Koulikoro	19	9.2	1.3	3	58.5	0.2
Ségou	59	2.9	4.0	133	1.3	9.1
Sikasso	6	27.1	0.4	2	68.5	0.2

**Table 5:** Mean aflatoxin concentrations (µg/kg) in maize, groundnut, and sorghum crops sampled from farmers’ fields in Niger across different regions. Same uppercase letters indicate crop means that are statistically similar in a column (i.e., across regions); same lowercase letters indicate means that are statistically similar in a row (i.e., across crops). Means of log aflatoxin concentrations were separated using Student–Newman–Keuls test (α = 0.05). ND: not detected. Niamey was not included in the analysis for sorghum because of the low number of samples.

	Total Aflatoxin (µg/kg)																	
	Maize						Groundnut						Sorghum					
Region	n	Min	Max	Mean	Median	Variance	n	Min	Max	Mean	Median	Variance	n	Min	Max	Mean	Median	Variance
Dosso	32	ND	5886	658.9 Aa	209.6	1.5 × 10^6^	40	ND	8593	627.5 Aab	72.7	2.1 × 10^6^	38	ND	1934	106.7 Aa	6.4	106,663
Zinder	40	ND	3721	276.1 Ba	ND	506,246	40	ND	7162	702.6 Aa	ND	2.5 × 10^6^	38	ND	1988	63.4 Bb	ND	104,206
Maradi	37	ND	924	99.5 Ba	ND	44,838	39	ND	5142	343.9 ABa	ND	848,354	39	ND	354	35.3 Ab	ND	6209
Tillabéri	14	ND	1368	210.6 Ba	ND	241,488	30	ND	1531	89.9 Ba	ND	93,912	30	ND	531	79.4 Aab	ND	20,391
Niamey	-	-	-	-		-	-		-	-		-	4	ND	655	258.7	ND	57,534

**Table 6:** Risk assessment of aflatoxin exposure from maize and sorghum crops samples from different regions in Niger. PDI: probable daily intake. MOE: margin of exposure. HCC rates: hepatocellular carcinoma rates (cancer/year/100,000 persons).

Region	Maize			Sorghum		
	PDI (ng/kg bw/day)	MOE	HCC Rates	PDI (ng/kg bw/day)	MOE	HCC Rates
Dosso	2100	0.1	119.7	706	0.2	40.2
Zinder	899	0.2	51.2	534	0.3	30.4
Maradi	310	0.5	17.7	253	0.7	14.4
Tillabéri	729	0.2	41.6	659	0.3	37.6
Niamey	-	-	-	2221	0.1	126.6

## Data Availability

All obtained data during the course of this study are available upon request from the corresponding authors.

## Statement of Compliance

In each country, the collections of samples of maize, groundnut, and sorghum were conducted complying with all relevant institutional, national, and international guidelines and legislation.
